# Exploring microbial dynamics in ferruginous caves: taxonomic and functional diversity across seasons and cave zones

**DOI:** 10.3389/fmicb.2025.1619203

**Published:** 2025-09-08

**Authors:** José Augusto Pires Bitencourt, Débora Marina Bandeira, Affonso Celso Gonçalves, Aline Snak, Danielly Cristina Marques de Castro, Rafaela de Lima Ribeiro, Leandro Araujo Argolo, Rafael dos Santos Scherer, Brenda Almeida Lima, Ulisses Brigatto Albino, Fabiana Gisele da Silva Pinto

**Affiliations:** ^1^Instituto Tecnológico Vale, Belém, Brazil; ^2^Rede de Biodiversidade e Biotecnologia da Amazônia Legal (BIONORTE), Instituto de Ciências Biológicas, Belém, Brazil; ^3^Graduate Program in Conservation and Management of Natural Resources, State University of Western Paraná, Cascavel, Brazil; ^4^Graduate Program in Energy Engineering in Agriculture, State University of Western Paraná, Cascavel, Brazil; ^5^Marabá Speleological Group, Marabá, Brazil; ^6^Graduate Program in Ecology and Natural Resources, Federal University of São Carlos, São Carlos, Brazil; ^7^Graduate Program in Chemistry, Federal University of Southern and Southeastern Pará, Marabá, Brazil

**Keywords:** cave microbiome, chemoheterotrophy, iron metabolism, metagenomics, microbial diversity, seasonal variation

## Abstract

Bacterial communities in ferruginous caves are known for their high diversity and functional adaptability to environmental conditions. In this study, we characterized the taxonomic and potential functional profiles of two iron-rich caves, GEM-1423 and GEM-1462, across photic, dysphotic, and aphotic zones during both rainy and dry seasons. High-throughput sequencing revealed distinct microbial community structures. GEM-1423 showed strong dominance of specific taxa, while GEM-1462 exhibited lower dominance of iron-metabolizing groups and higher beta diversity, particularly in the dry season—indicating a greater degree of species replacement. Notably, iron availability emerged as a key factor influencing microbial dynamics in both caves, affecting community composition and functional pathways. Core genera, such as *Bacillus*, *Acidothermus*, *Mycobacterium*, and *Acidisphaera,* were associated with nitrogen and carbon cycling, as well as indirect iron solubilization through production of organic acids. Potential functional profiles varied seasonally: energy metabolism was enriched during the dry season, while nutrient cycling pathways were more abundant in the rainy season. We also detected taxa involved in manganese oxidation, urea degradation, and functions with biotechnological relevance, including antimicrobial compound production and metal resistance. These findings highlight the complex interactions between environmental factors, microbial diversity, and ecosystem function in ferruginous caves, and underscore the biotechnological potential of microbial communities from extreme environments.

## 1 Introduction

Subterranean environments such as caves are defined by low temperatures, high humidity, and nutrient-poor conditions, making them challenging habitats for microbial life (Howarth and Moldovan, 2018). Despite these constraints, caves host diverse microbial communities and represent valuable reservoirs for the discovery of novel bioactive compounds (Gatinho et al., 2023). The unique combination of mineralogical features, stable microclimates, and ecological isolation within caves creates favorable conditions for the biosynthesis and accumulation of biologically active molecules (Farda et al., 2022). Exploring these environments as sources of natural products highlights their potential as living laboratories for drug discovery and biotechnological innovation (Ghosh et al., 2017; Rangseekaew and Pathom-Aree, 2019; Zada et al., 2021).

Caves harbor unique underground communities of organisms and microclimates, often supporting dense populations of extremophiles and exhibiting high microbial diversity. Microorganisms adapted to cave environments typically exhibit slow metabolic and growth rates (Epure et al., 2014). Studying microbial communities in cavernous ecosystems is crucial as these microorganisms play a vital role in maintaining environmental equilibrium (Bardgett and van der Putten, 2014; Muhammad and Saadu, 2023; Philippot et al., 2021). Their survival strategies, adapted to extreme conditions, encompass diverse mechanisms, from versatile metabolism thriving in nutrient scarcity to symbiotic partnerships bolstering resilience (Iqbal et al., 2023; Muhammad et al., 2024). Exploring these communities reveals their survival tactics and offers insights into broader ecosystem functioning, emphasizing the interconnectedness within cavernous environments (Krause et al., 2014; Shrestha et al., 2021).

Cave microbiomes typically comprise microorganisms, including Bacteria, Archaea, Fungi, and occasionally algae (Protists) and viruses (Mkwata et al., 2021). Bacteria often dominate cave walls and speleothems, even in environments apparently devoid of organic matter (Lavoie et al., 2017) influencing processes such as speleogenesis and mineral weathering (Cuevza et al., 2009). The composition and functional profiles of microbial communities in cave ecosystems significantly influence biogeochemical cycles and secondary metabolite production (Gabriel and Northup, 2013; Kosznik-Kwaśnicka et al., 2022; Ma et al., 2021; Ortiz et al., 2014) with potential benefits for humans and ecosystem balance. Moreover, in some caves these communities may also serve as primary producers sustaining trophic webs (Barton and Northup, 2007).

These microbial communities are pivotal in modifying mineral dynamics by engaging in processes like mineral weathering and dissolution, consequently shaping the availability of vital nutrients (Nicolitch et al., 2017; Wild et al., 2022). This intricate association between microorganisms and minerals not only contributes to the physical structure of cave ecosystems but also regulates chemical environments conducive to synthesizing a diverse array of secondary metabolites (Mandal et al., 2017). Microbial metabolic activities, often intricately linked with mineral substrates, facilitate biochemical reactions that hold promise for generating bioactive compounds (Bogdan et al., 2023; Gatinho et al., 2023).

Understanding the interactions between microbial functions, mineral availability, and secondary metabolite biosynthesis is crucial for comprehending cave ecosystem dynamics and exploring novel biotechnological and pharmaceutical applications (Ferreira et al., 2023; Figueiredo Silva et al., 2013; Zada et al., 2021). To clarify the role of cave microbial communities in biogeochemical cycles and identify potential sources of bioactive metabolites (Kosznik-Kwaśnicka et al., 2022; Zhu et al., 2022), it is essential to characterize their structural, compositional, and functional profiles within these extreme environments. Metabarcoding has emerged as an effective approach for elucidating complex microbial communities and their ecological roles (Wani et al., 2025), including in challenging habitats such as caves (Wani et al., 2022). A key factor influencing microbial communities in caves is the natural gradient of light availability, which defines three distinct ecological zones: photic, dysphotic, and aphotic (Hershey and Barton, 2018). Although light availability diminishes progressively toward deeper cave regions, seasonal variations in temperature, humidity, and nutrient input continue to influence microbial dynamics, even within the aphotic zone. These combined environmental gradients shape microbial community structure and function, reflecting a complex interplay between ecological adaptation and environmental factors. Caves also present different lithologies, and sediments are a valuable reservoir for metabolites produced by the microbial communities inhabiting the ecosystem (Epure et al., 2014; Rangseekaew and Pathom-Aree, 2019; Tomczyk-Żak and Zielenkiewicz, 2016). Iron ore caves, for example, may represent a resource-limited habitat as they present extreme conditions and unique characteristics when compared to other caves of different lithologies. Iron ore caves are composed of 90% iron oxides, very acidic soil, with low fertility rates and high temperatures that reach almost 70 °C on the surface (Mota et al., 2015). In iron caps and iron formations, the associated geo-environmental and biological heritage remains poorly understood (Carmo and Kamino, 2015), and the microbiological potential within these habitats is also misunderstood.

In Brazil, caves exhibit diverse lithologies reflecting the country’s complex geological history. The Carajás region, located in Pará state, hosts the largest concentration of natural underground cavities in Brazil, with over 2,800 documented caves and many still unexplored (Lima et al., 2023). These caves predominantly occur in iron-rich lithologies, characterized by low permeability. Unlike carbonate caves, iron-rich caves may directly influence microbial metabolism by favoring microorganisms capable of reducing iron and other metals as terminal electron acceptors (Auler et al., 2019). Furthermore, this region is marked by extreme environmental conditions, including nutrient-poor soils, high concentrations of heavy metals (e.g., iron, aluminum, manganese), elevated humidity (~90%), temperatures between 34 °C and 38 °C, and pronounced seasonal rainfall patterns 2,000 to 2,400 mm annually (Barros et al., 2020).

The Campos Ferruginosos National Park (CFNP), a conservation unit within the Carajás region, aims to safeguard speleological heritage (Barros et al., 2020). Despite their ecological and biotechnological significance, the microbial communities of the ferruginous caves in the CFNP remain largely unknown. These unexplored environments may represent an important reservoir of microbial biodiversity, potentially valuable for biotechnological innovation and ecological conservation (Kosznik-Kwaśnicka et al., 2022; Reboleira et al., 2022).

Although caves represent critical ecosystems, their microbial diversity, especially under extreme conditions such as those in ferruginous environments, is poorly understood in South America (Barros et al., 2020). In this context, we aim to characterize microbial communities within two ferruginous caves in the CFNP, differing in iron concentrations and lithological features (jaspilite/canga vs. jaspilite). We evaluate taxonomic and functional diversity across ecological zones (photic, dysphotic, and aphotic) and seasons (rainy and dry) to understand how environmental factors shape microbial community structure and function.

## 2 Materials and methods

### 2.1 Sampling description

**Figure 1:**
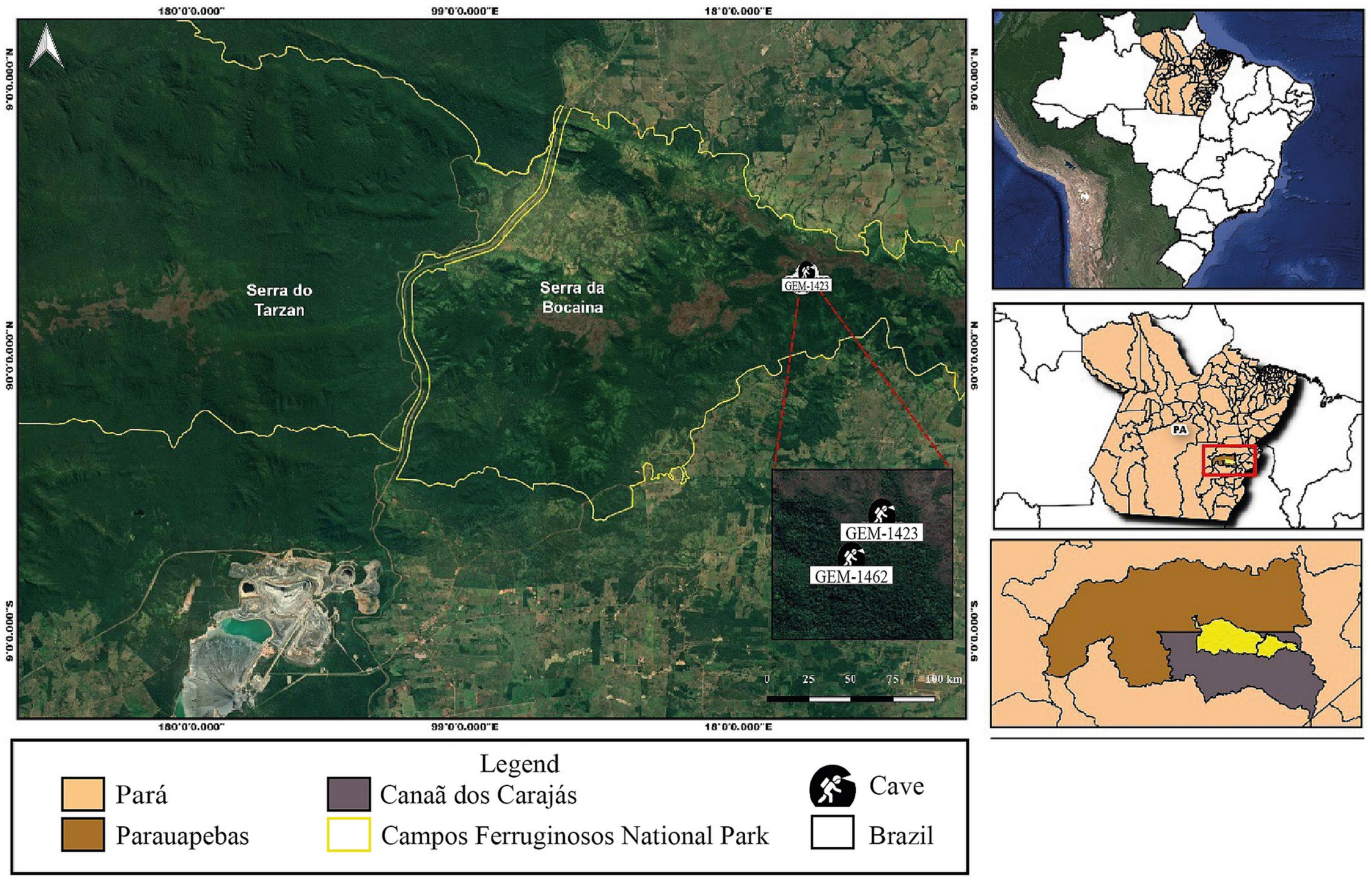
Location of studied caves GEM-1423 and GEM-1462 in Serra da Bocaina, Campos Ferruginosos National Park, Pará, Brazil.

The study site was in Serra da Bocaina, within the Campos Ferruginosos National Park (CFNP), in the municipality of Canaã dos Carajás, Pará. Based on secondary data from the Diagnosis and Relevance Analysis Report of 235 caves in Serra da Bocaina (Piló et al., 2023), the caves GEM-1423 (6° 18′ 49.27″ S, 49° 53′35.06″ W) and GEM-1462 (6° 18′ 45.29″ S, 49° 53′ 41.71″ W), Figure 1, were selected considering the relevance, lithology, morphology, hydrology, presence or absence of organic matter and presence of soil, information indicated on the topographic map, geo and geo-biospeleology sheet. The lithology of cave GEM-1423 is characterized by jaspilite/canga and GEM-1462 by jaspilite. The research was conducted under the license of the Biodiversity Authorization and Information System (SISBIO) No. 79255-1/22 e 86153-1/22 and Genetic Heritage Management System (SISGEN) No. A13D56A/22.

In both caves, the internal area was classified into three zones, following Trajano and Bichuette (2006): (1) photic zone at the cave entrance (high level of light); (2) dysphotic zone (intermediate level of light); and (3) aphotic zone (absence of light). Soil samples were collected from the surface layer of the cave, within the first 10 cm, because this zone typically exhibits higher microbial activity, organic matter input, and more responsiveness to environmental changes, making it a key indicator of microbial community dynamics (Fierer et al., 2003; Bahram et al., 2018). Quantities used ranged from 100 g to 500 g and sampling was conducted at three points in each zone, resulting in a total of nine sampling points. The samples were placed in identified, sterile plastic bags (Ziplock), with the help of a sterile gardening shovel that was disinfected after each collection with 70% alcohol. The samples were then stored in a thermal box at 4 °C and transported to the laboratory for analysis.

### 2.2 Environmental and soil chemical characterization of caves

Physical and geographical variables related to the caves, such as lithology, surface area, internal volume, slope, elevation, and horizontal projection, were compiled from Piló et al. (2014). For the analysis of soil chemical composition, samples were manually disaggregated and sieved using a 2 mm mesh. The resulting fine earth fraction was oven-dried at 40 °C in a forced-air circulation oven and then stored in properly labeled containers following standardized procedures (Teixeira et al., 2017).

Soil pH was measured in a 0.01 mol L^−1^ CaCl₂ solution (Reatec^®^, Reagen, Brazil), using a soil-to-solution ratio of 1:2.5 (m/v), according to the methodology described by Teixeira et al. (2017). pH readings were performed using a digital pH meter (Phox^®^, model P1000), previously calibrated with standard buffer solutions at pH 4.0 and 7.0 (Merck^®^, Darmstadt, Germany). Total concentrations of Co, Ca, Cu, Ni, Mn, Mg, Na, Pb, Zn, and Fe were determined by acid digestion using a nitric-perchloric acid mixture (HNO₃:HClO₄) in a 3:1 volumetric ratio (Reatec^®^, Reagen, Brazil), following the protocols established by the Association of Official Analytical Collaboration (AOAC, 2023). For each sample, exactly 500 mg of oven-dried soil (dried at 40 °C) were weighed using an analytical balance (Shimadzu^®^, model AY220) and transferred to 125 mL digestion tubes. Then, 8 mL of the acid mixture was added. The samples underwent a pre-digestion step at room temperature for 3 to 4 h to promote the initial breakdown of the organic and mineral matrix. Following pre-digestion, the tubes were gradually heated in a micro-digestion block (Tecnal^®^, model TE-040/25) until reaching 120 °C, allowing controlled release of nitrogen dioxide (NO₂) vapors. The temperature was then raised to 200 °C and maintained until complete evaporation of perchloric acid vapors, ensuring total oxidation of the remaining organic matter. The total digestion time was approximately 6 to 8 h. At the end of the digestion process, the tubes were cooled to room temperature, and the resulting material was resuspended in 25 mL of distilled and deionized water. Element quantification was performed using flame atomic absorption spectrophotometry (FAAS) with a GBC spectrophotometer (model SavantAA), under optimized operational conditions for each element analyzed, as per procedures established by Teixeira et al. (2017).

### 2.3 DNA extraction, 16S rRNA gene amplification and sequencing analysis

To identify the microbial community composition in the two caves across different zones, and seasons, DNA was extracted from 0.25 g of soil samples using the QIAGEN PowerSoil^®^ DNA Isolation Kit (QIAGEN, Hilden, Germany) after homogenization in a vortex. The library construction was carried out following Illumina’s 16S Metagenomic Sequencing Library Preparation protocol (Illumina, San Diego, CA, United States). To analyse the taxonomic composition of the microbial communities, the V3–V4 regions of the 16S ribosomal gene were amplified through polymerase chain reaction (PCR) using universal primer pairs S-D-Bact-0341-b-S-17-N and S-D-Bact-0785-a-A-21-N (Klindworth et al., 2013). The PCR mixture and amplification program followed Silva et al. (2024). Subsequently, unique sequence adapters (indexes/barcodes) were added to each sample through the PCR Index step using the indexes from the Nextera XT Library Preparation Kit, and the sequencing run was performed on the Illumina MiSeq platform using MiSeq V3-600 cycles kit. For the identification of the molecular signatures of the bacterial communities present in the soil samples, the raw sequences were subjected to a PIMBA-PIpeline for MetaBarcoding Analysis (Oliveira et al., 2021), based on the QIIME (Quantitative Insights into Microbial Ecology) pipeline (Caporaso et al., 2010). To improve the quality of the metabarcoding, all sequences shorter than 100 bp were discarded. Sequences were then assigned into Amplicon Sequence Variants (ASV) using Swarm 2 (Mahé et al., 2015). The taxonomy identification of ASVs was performed by comparing them with sequences available in the SILVA132 database (Quast et al., 2012). Mitochondrial and chloroplast DNA sequences were removed from the analysis, and all unidentified microorganisms in this database were grouped as uncultured at the matching taxonomic level. Microbial analyses were performed in phyloseq (McMurdie and Holmes, 2013) and microbiome packages (Lahti et al., 2019).

We conducted an imputed metabarcoding analysis through genome prediction to infer metabolic activity with FAPROTAX v1.2.6 (Louca et al., 2016) using ASV relative abundance of taxa. Functional Annotation of Prokaryotic Taxa (FAPROTAX) is a database that extrapolates the functions of cultured prokaryotes to estimate their metabolic and ecologically relevant roles in nature (Picazo et al., 2021). The functional profiles presented here should be viewed as proxies for microbial metabolism function, offering ecological insight. It is particularly suitable for annotating and predicting biogeochemical cycles involving elements such as carbon (*chitinolysis*, *cellulolysis*, *aromatic_hydrocarbon_degradation*, *aromatic_compound_degradation, aliphatic_non_methane_hydrocarbon_degradation*, *hydrocarbon_degradation*, *nonphotosynthetic_cyanobacteria*, *chemoheterotrophy*), nitrogen (*ureolysis, aerobic_nitrite_oxidation*, *nitrification*, *nitrite_respiration*, *nitrate_ammonification*, *nitrite_ammonification*, *aerobic_ammonia_oxidation*, *nitrate_respiration*, *nitrogen_respiration, nitrate_reduction*), manganese (*manganese_oxidation*), and sulfur (*sulfate_respiration*, *respiration_of_sulfur_compounds*). Metabolic processes linked to the utilization of organic and inorganic chemical substances as energy sources, as well as the assimilation of organic and inorganic compounds as primary carbon sources, were categorized as energy functions (*aerobic_chemoheterotrophy*, *chemoheterotrophy* and *fermentation*). Additionally, parasitism or symbiosis categories were classified as a pathogenic function (*predatory_or_exoparasitic*, *human_pathogens_all*, *invertebrate_parasites*, *human_associated* and *animal_parasites_or_symbionts*).

### 2.4 Statistical analysis

To conduct data analyses, the raw data was refined by calculating correspondent relative abundances before using them as input variables. The Shannon-Weiner index (H) was calculated to estimate ASVs richness and abundance from all samples. Then the Wilcoxon test was applied to compare differences in observed and estimated ASVs richness between the two caves (GEM-1423 and GEM-1462) and the annual seasons (dry and rainy), and Kruskal–Wallis test for the cave’s zones (photic, dysphotic and aphotic). The same approach was used to compare the metabolic diversity index present in each cave, zone, and season. In this, the Shannon–Weaver index was used to measure the metabolic diversity index and calculated using results from the prediction of metabolism activity generated by FAPROTAX (Picazo et al., 2021). Also, beta regression model (*betareg*) was used to explore changes in the composition of bacteria colonizing the cave substrate using the variable groups light zones and season.

To understand the environmental characteristics of the two caves, considering the temporal variation between dry and rainy seasons and between cave zones, we performed a Principal Component Analysis (PCA) using the *FactoMineR, prcomp*, and stats packages in R. The PCA included physical and geographical variables of the caves (lithology, area, volume, slope, height, horizontal projection) and soil-chemical composition (Co, Ca, Cu, Ni, pH, Mn, Mg, Na, Ca, Pb, Zn, and Fe). All numerical data were standardized by applying the *hellinger* method for values with a mean equal to zero with the *decostand* function from the *vegan* package (Borcard et al., 2011) in R.

To test differences in the community composition among caves, zones, and seasons, we performed a Permutational Multivariate Analysis of Variance using the *adonis2* function in *vegan* package (PERMANOVA) based on the Euclidean distance metric of genus identity among the whole communities. To further investigate the variation in the composition of communities through their relative abundances according to abiotic characteristics, we performed a redundancy analysis (RDA). We used the full set of 21 abiotic and environmental variables related to the communities and carried out a forward selection to select the partial set of variables with significant effects on community composition, considering the higher adjusted R^2^ model’s value. To minimize weak correlations and enhance the interpretation of community composition in relation to environmental variables, we included the 50 most abundant genera from each community in the Redundancy Analysis (RDA). After 10 steps, the best-fitted model (adjusted R^2^ = 0.45, see results) included nine selected variables: height, lithology, Fe, Co, Cu, Mg, Na, Ca and Pb. To perform the RDA, the abiotic and environmental variables were standardized with a mean equal to zero using the *standardize* method of the *decostand* function in the *vegan* package; and the environmental variables were homogenized by standardizing the values using the *hellinger* method, also from the *decostand* function (Borcard et al., 2011). The redundancy model was constructed using the *rda* function in the *vegan* package (Oksanen et al., 2013) and the significance of the environmental variables was tested using the *anova.cca* function (Legendre et al., 2011). All analyses were conducted in R software version 4.1.2 (R Core Team, 2021) and considered *p*-values <0.05 as statistically significant.

## 3 Results

### 3.1 Taxonomic composition of bacterial community in ferruginous caves

The 16S rRNA dataset (V3–V4 region) comprised 7,365,020 reads, with the number of sequences from each sample ranging from 43,804 to 463,723 reads. These sequences were grouped into 805 ASVs. After cleaning the sequences and removing mitochondrial and chloroplast-associated sequences, 522 ASVs remained, of which 422 were identified as Bacteria.

**Figure 2:**
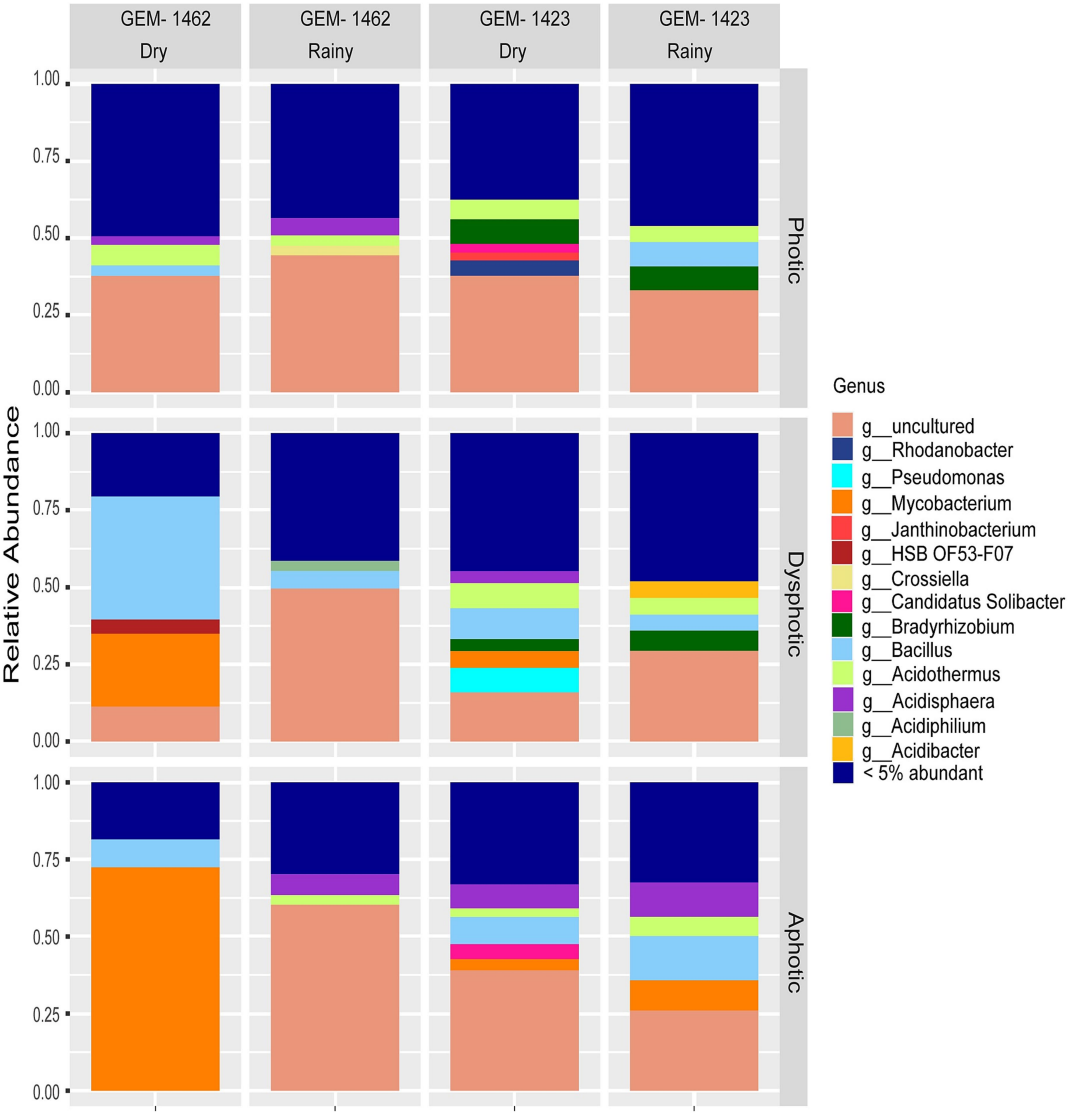
Relative abundance of bacterial genera in photic, dysphotic, and aphotic zones of iron-rich caves GEM-1462 and GEM-1423 during the dry and rainy seasons.

A total of 164 microbial taxa were identified across both caves, with 80% of the taxa shared between them. Cave GEM-1423 harboured 28 exclusive taxa (14%), while GEM-1462 presented 14 unique taxa (7%). Taxa distribution also varied according to the light gradient. A majority of the taxa (97 taxa; 65%) were present in all three zones. In contrast, 13 taxa (9%) were exclusive to the dysphotic zone, while only three taxa (2%) were exclusive to either the aphotic or photic zones (Figure 2). Overall, the microbial community was predominantly composed of Bacteria, accounting for 99% of all identified taxa. A total of 27 phyla were identified in both caves, including WPS-2, Verrucomicrobia, Thaumarchaeota, TA06, Spirochaetes, Rokubacteria, Proteobacteria, Planctomycetes, Patescibacteria, Nitrospirae, Latescibacteria, Gemmatimonadetes, GAL15, Firmicutes, FCPU426, Euryarchaeota, Elusimicrobia, Diapherotrites, Dependentiae, Cyanobacteria, Crenarchaeota, Chloroflexi, Chlamydiae, Bacteroidetes, Armatimonadetes, Actinobacteria, and Acidobacteria, all detected in sediment samples from both caves [Figure 2, see Supplementary Figure 1 for relative abundances at the phylum (A) and family (B) levels].

Despite observing 100 unique genera, core taxa observed in caves are comprised of *Bacillus*, *Acidothermus*, *Mycobacterium*, *Acidisphaera*, *Bryobacter*, *Candidatus Solibacter*, *Bradyrhizobium*, *Acidibacter* (using a threshold of 15%, Supplementary Figure 2). When examining the relative abundance data for each cave, it was observed that in cave GEM-1423, *Bacillus*, *Acidisphaera*, *Mycobacterium*, *Pseudomonas*, and *Bradyrhizobium* were some of the most expressive genera identified across different seasons and zones (Figure 2). During the dry season, *Bacillus* accounted for 10% of the total bacterial community in the dysphotic zone. In turn, in the rainy season, *Bacillus* relative abundance decreased in the dysphotic zone to 5.15%, and increased in the aphotic zone to 14.33%. *Acidisphaera* accounted for 11.20% of the total relative abundance of the aphotic zone during the rainy season and 15.68% in the dry season. *Mycobacterium* showed varying abundances across seasons and zones in cave GEM-1423, representing 10% in the aphotic zone during the rainy season and 7.26% in the dry season, with a lower relative abundance in the dysphotic zone during the dry season (5.47%). *Pseudomonas* was only detected in high abundance (15.84%) in the dysphotic zone during the dry season. *Bradyrhizobium* was identified with a relative abundance of 7.8% in photic zone in both seasons (dry and rainy) and at 7.73% in the dysphotic zone dry season. Some minor groups represented in GEM-1423 were *Acidibacter* (5.42%) found in the dysphotic zone during the rainy season and *Candidatus solibacter* (9.70 and 0.57%) detected in the aphotic and photic zones during the dry season.

As for the cave GEM-1462, the most expressive abundances encompassed the genera *Bacillus*, *Acidothermus*, *Acidisphaera*, and *Mycobacterium* (Figure 2). *Bacillus* was predominantly observed during the dry season, particularly in the dysphotic zone, with 44.34% of the total relative abundance. During the rainy season, *Acidothermus* was found in the photic zone at 67.77% and in the aphotic zone at 62.30%. During the dry season, *Acidothermus* was observed in the photic zone at a concentration of 8%. Additionally, *Acidisphaera* was present at 6.90% in the aphotic zone during the rainy season in GEM-1462. The relative abundance of *Mycobacterium* was a bit lower in the dry season’s photic zone (5.62%) compared to the rainy season’s aphotic zone at 6.90%. Other genera with lower abundances in GEM-1462 included *Acidiphilium* (6.55%) in the dysphotic zone during the rainy season, and HSB OF53-F07 (0.91%) in the dysphotic zone during the dry season.

### 3.2 Bacterial diversity across caves, zones and seasons

**Figure 3:**
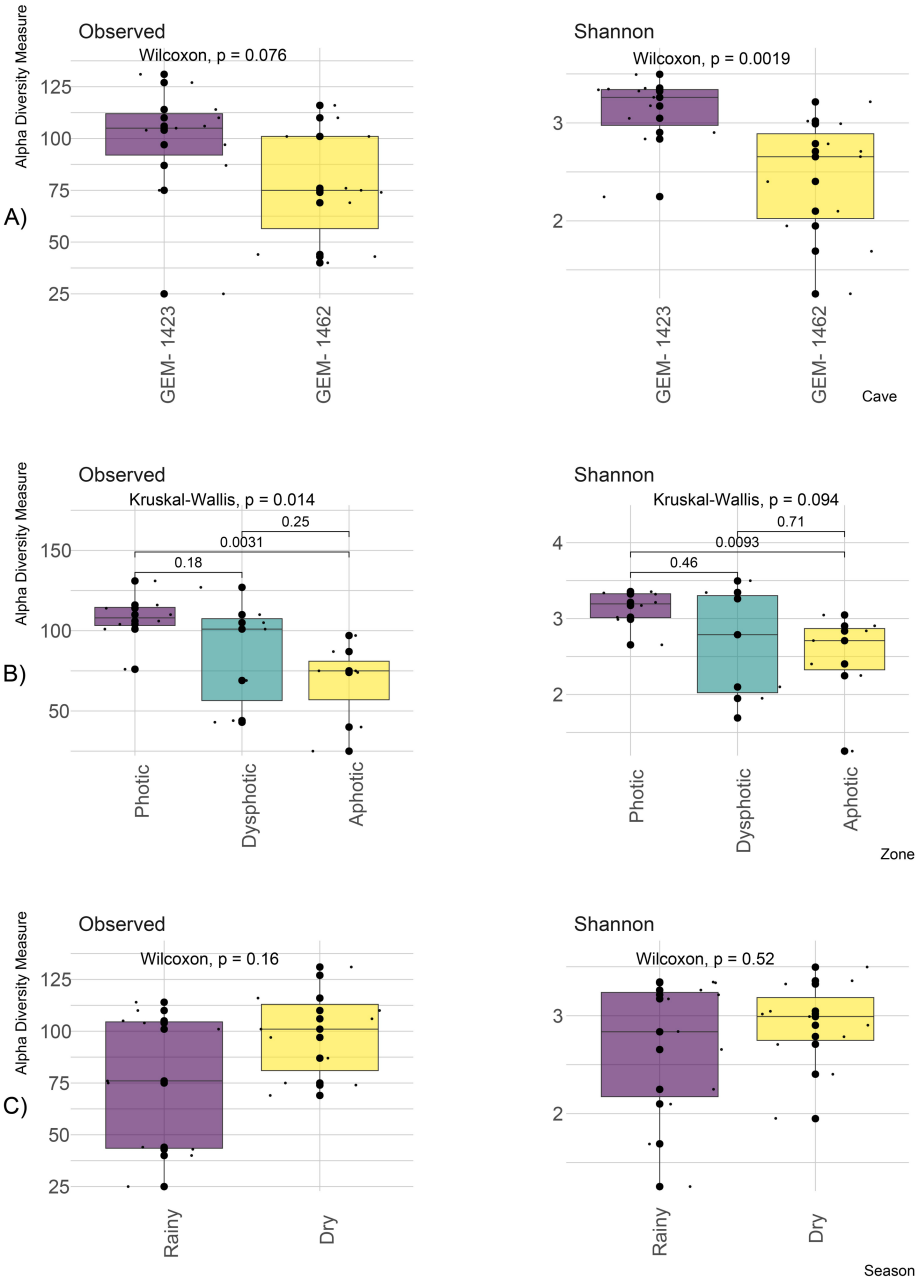
Alpha diversity showing the observed richness of genus and Shannon diversity index between both GEM-1423 and GEM-1462 caves **(A)**; zones according to light availability: photic, dysphotic and aphotic **(B)**; rainy and dry seasons **(C)**.

The richness of genera did not differ between the two caves (Wilcoxon, *p* = 0.076), but Shannon diversity was higher in GEM-1423 (Wilcoxon, *p* = 0.001; Figure 3). In general, GEM-1423 showed higher variability of genus richness (98.3 ± 29.1; 3.12 ± 0.35) and genus equitability (Shannon diversity index) in comparison with GEM-1462 (77.2 ± 27.3; 2.43 ± 0.62 mean ± SD genus richness; Shannon diversity index) (Figure 3A).

Genus richness along the photic gradient considering both caves showed differences (Kruskal–Wallis, *p* = 0.014), with greater richness observed in the photic zone (107 ± 15.7) compared to the dysphotic (85.6 ± 33.5) and aphotic zones (67.6 ± 25.7) (*p* < 0.001; Figure 3B). There was a tendency for Shannon diversity to be higher in the photic zone (3.13 ± 0.24) compared to the aphotic zone (2.49 ± 0.6) (Kruskal–Wallis, *p* = 0.094; Figure 3B), showing a higher dominance of genera in the aphotic zone. The dysphotic zone exhibited greater variability in genus richness and Shannon diversity compared with the photic and aphotic zones (Figure 3B).

The annual dry and rainy seasons showed no significant differences in genus richness or Shannon diversity (Wilcoxon, *p* = 0.16 and *p* = 0.52, respectively), although genus diversity was more variable in the rainy seasons (Figure 3C). Genus richness in dry season was 99.4 ± 21.3 and 76.1 ± 33 in the rainy season. Shannon diversity was 2.91 ± 0.45 and 2.65 ± 0.73 for dry and rainy seasons, respectively.

**Figure 4:**
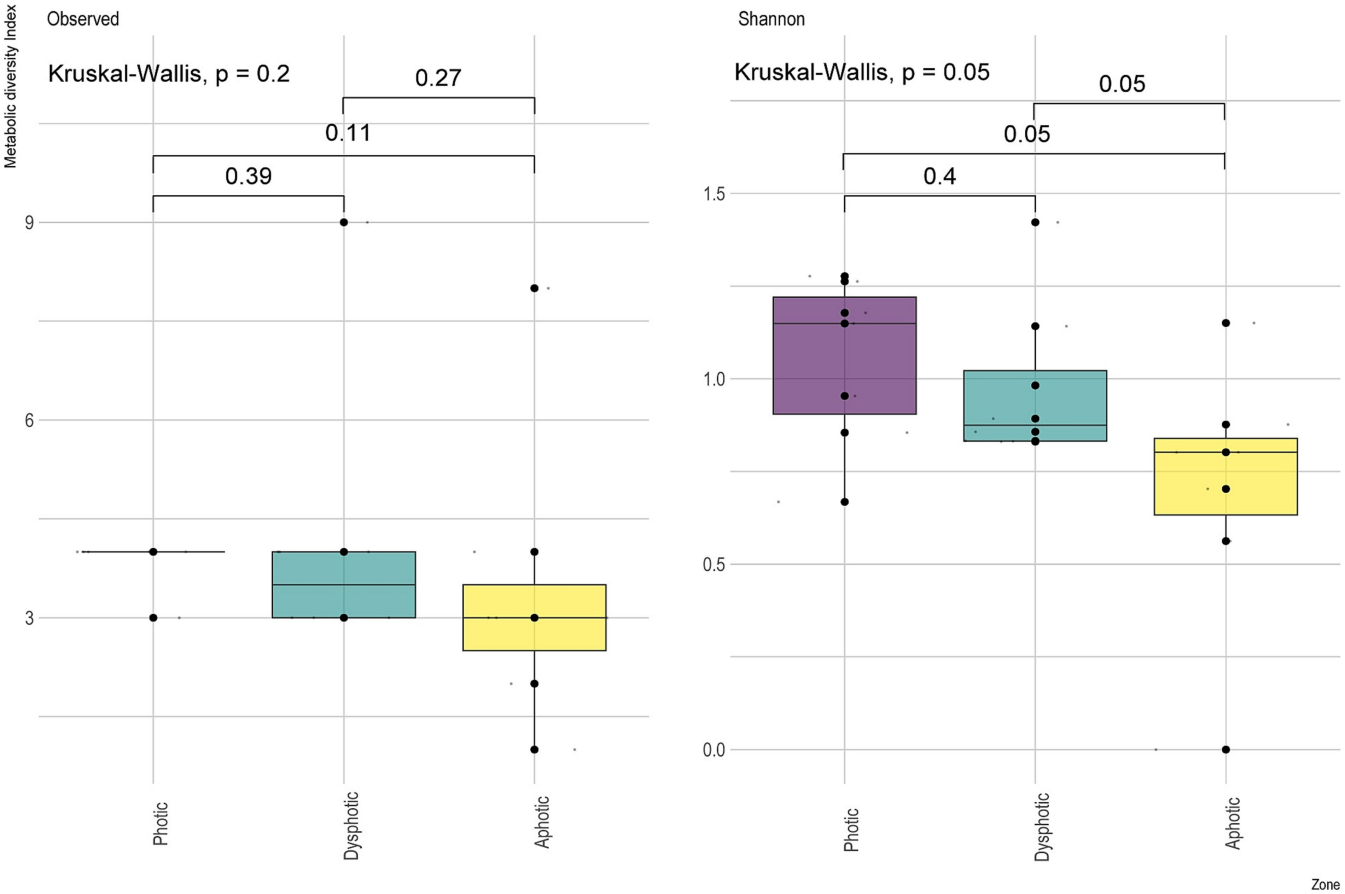
Metabolic diversity index showing the observed richness of predicted physiological functions and Shannon diversity index between the sampled light zones from GEM-1423 and GEM-1462 caves.

The metabolic diversity index (Supplementary Figure 3; Figure 4) observed Richness among the light incidence zones in the caves did not differ significantly (Figure 4), although Shannon diversity was significant, and the *post hoc* test indicated that differences for the functions between the photic and dysphotic zones were marginally significant.

### 3.3 Relationship between bacterial community and soil properties between caves

**Figure 5:**
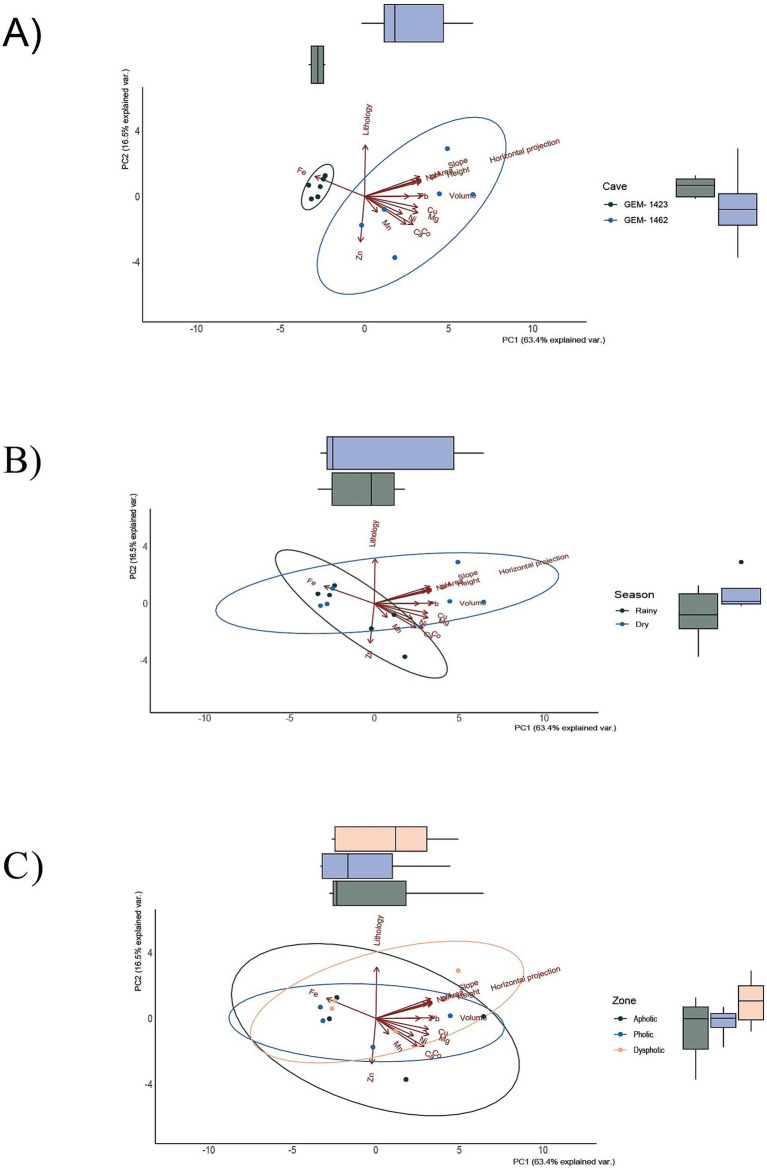
Principal component analysis (PCA) according to two caves, GEM-1423 and GEM-1462 **(A)**, dry and rainy seasons **(B)**, and light availability of three different cave zones **(C)**. Each data point represents a replicate sample of communities.

PCA was used as an exploratory method for data visualization and for distributing bacterial genera according to environmental variables, including caves, season, and zoning based on light. Axis 1 of the PCA explained 63.4% of the variation in environmental characteristics between GEM-1423 and GEM-1462 (Figures 5A–C). The main difference between the caves lies in their relationship with iron concentrations, with GEM-1423 being more closely associated with the presence of this mineral. GEM-1462 is more closely related to the concentrations of other nutrients (Figure 5A). The PCA’s second axis explained 16.5% of the variation in environmental and soil characteristics, with sample discrimination driven mostly by differences in lithology and Zn composition. Considering the different zones of the caves and the annual seasons, there are no strong indications of differentiation in the composition of the caves (Figures 5A,C). However, comparing the characteristics of the caves between the dry and rainy seasons, there seems to be more variation related to iron and zinc in the rainy season than in the dry season (Figure 5B).

**Figure 6:**
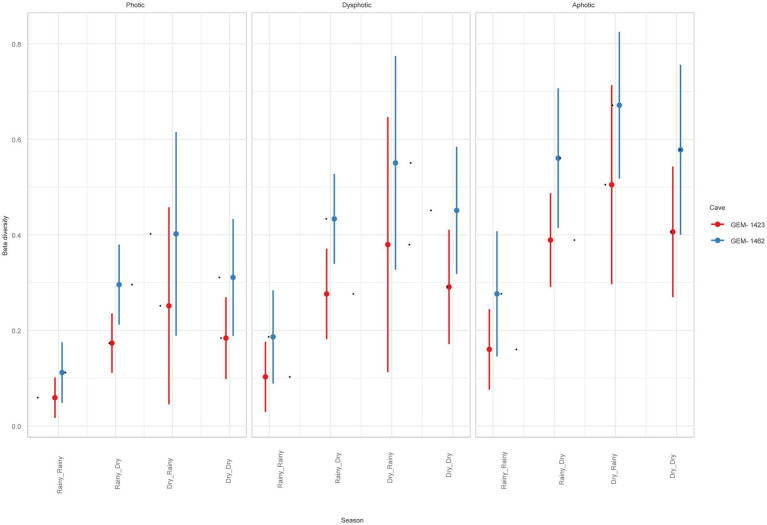
Beta diversity comparison between studied caves, light zones, and seasons.

Beta diversity was higher for GEM-1462 than GEM-1423 in all observed environmental variables (season and light zone, *R*^2^ = 0.61, and *p* < 0.05). It is approximately 20% higher in cave GEM-1462 during the dry season (Figure 6) than in GEM-1423 during the rainy season (photic, dysphotic, and aphotic zones). The cave GEM-1462 exhibits more taxon exchange in the dysphotic and aphotic zones than in the photic zone, with these changes in composition possibly occurring between these two zones or in response to the external environment.

**Figure 7:**
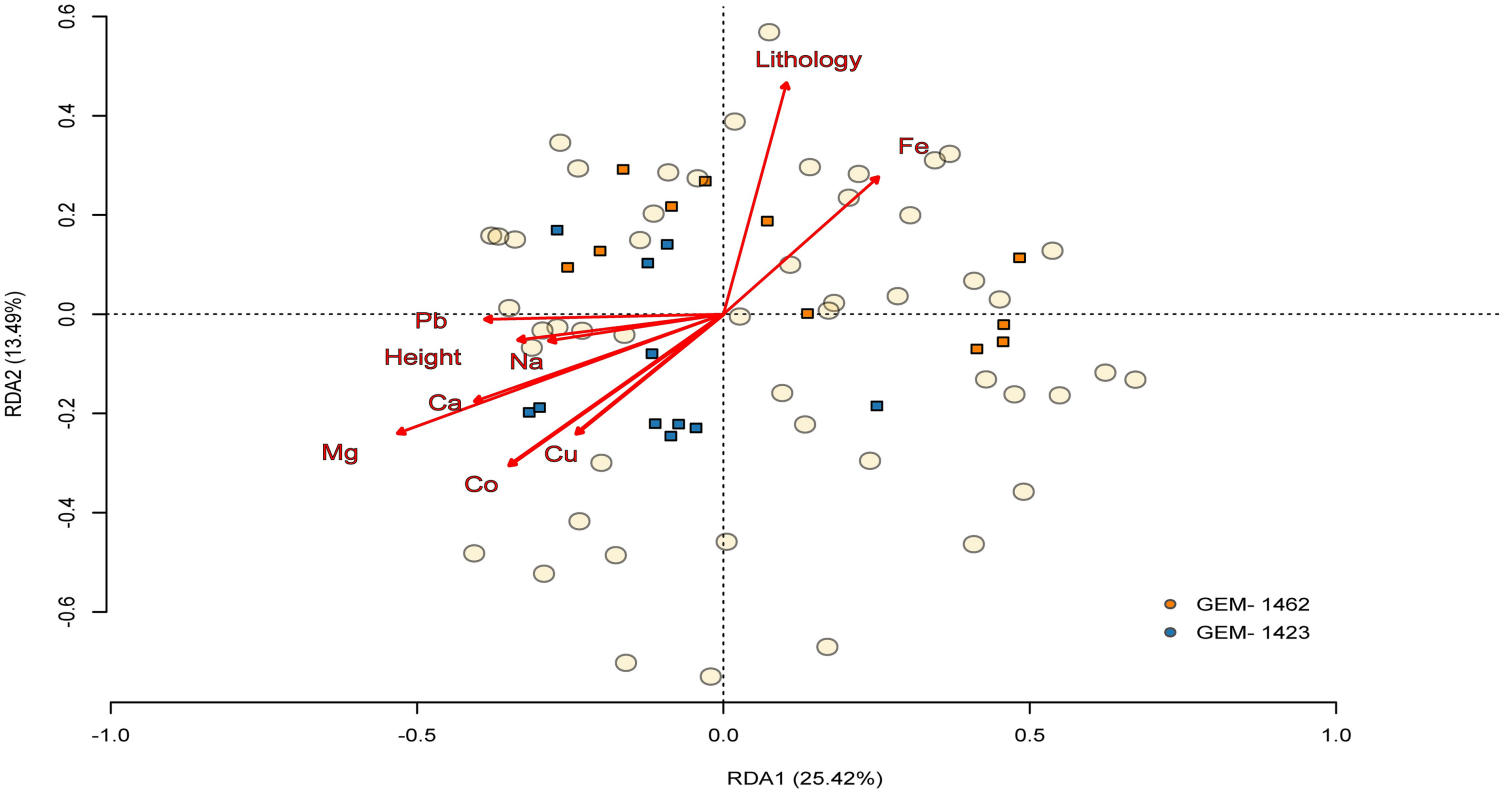
Triplot of the relationship between abiotic/environmental variables and the community’s composition between the two caves (GEM-1423 and GEM-1462). Composition is made from the 50 most abundant genera, and circles and squares represent, respectively, the genus composition of communities and abiotic/environmental samples, both according to the ordination axes 1 and 2 extracted from redundancy analysis. The arrows indicate the orientation of caves composition based on the abiotic/environmental variables.

Both caves exhibited a variable set of correlations between the relative abundance of certain genera, with the following three genera standing out in terms of significance: zinc, sodium, and iron (Supplementary Figure 4). The communities’ species composition varied between caves (PERMANOVA tests: F1, 20 = 3.485; *R*^2^ = 0.148; *p* = 0.005) and between seasons (F1, 20 = 3.661; *R*^2^ = 0.155; *p* = 0.005), but not among the cave zones (F1, 20 = 0.991; *R*^2^ = 0.047; *p* = 0.399). The environmental characteristics explained 43.82% (constrained) of the variation in genus community composition between the caves (RDA model: F11, 10 = 3.695; *p* < 0.001, Figure 7). For the seasons, the environmental variables explained 73.65% of changes in genus community composition. The most important abiotic/environmental characteristics to explain changes in cave composition were, by order, Mg, Cu, height, lithology, Ca, Pb and Fe (see more details in Table 1; Supplementary Table 1 with chemical and environmental variables data and Supplementary Figure 4 with correlation figure).

**Table 1:** Permutation test for constrained correspondence analysis showing the analysis of variance of all environmental variables included on RDA models.

Abiotic/environmental variables	df	Variance	*F*	*p*-value
Mg	1	0.186	7.783	**<0.001**
Lithology	1	0.110	4.605	**<0.001**
Height	1	0.111	4.657	**<0.001**
Na	1	0.065	2.723	**0.006**
Co	1	0.027	1.136	0.309
Ca	1	0.072	3.025	**0.003**
Pb	1	0.059	2.478	**0.013**
Cu	1	0.113	4.739	**<0.001**
Fe	1	0.051	2.392	**0.009**
Residual	12	0.286	—	—

The degree of freedom (df), explained variance, statistical *F*-values, and *p*-values with their respective significance in bold are reported.

### 3.4 Functional prediction using FAPROTAX

**Figure 8:**
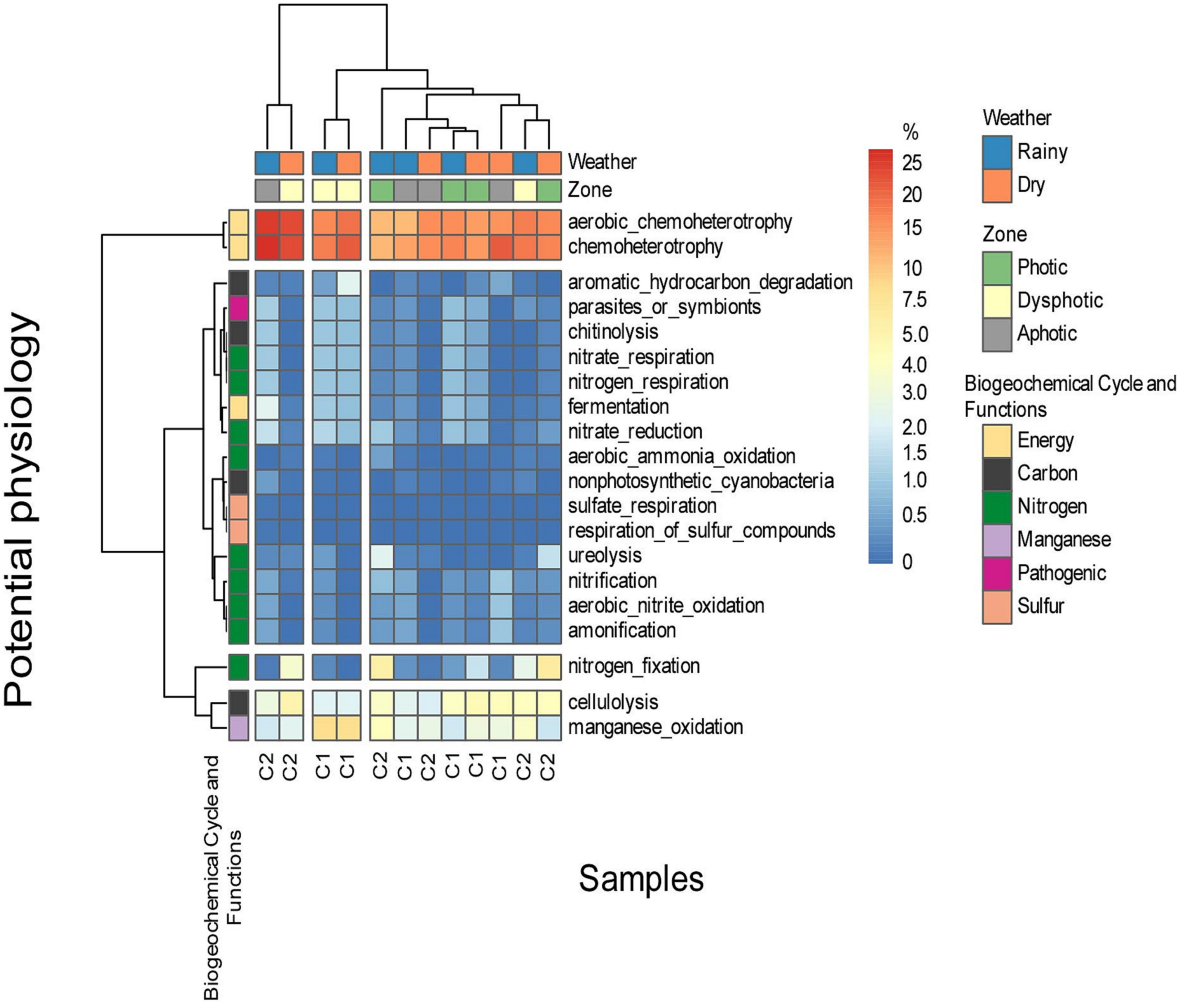
The prediction of the potential physiology of microorganisms found on soil samples of both caves, in different zones, and under different seasons. C1 represents cave GEM-1423 and C2 represents cave GEM-1462.

A high representation of energy metabolism was detected among the physiological potential of the ASVs identified in the caves, particularly related to carbohydrate metabolism, cellulose degradation, nitrogen metabolism, metabolism dependent on oxygen, aerobic chemoheterotrophy and manganese metabolism (Figure 8).

Most observed potential metabolic functions were associated with nutrient cycling, particularly nitrogen and carbon. Additionally, we recognized significant pathways dedicated to energy production. In GEM-1423, during the rainy season and in the aphotic zone, about 25% of bacteria showed a potential metabolism of chemoheterotrophy, and basically, were dependent on oxygen as a final electron acceptor. However, during the dry season, the proportion of microorganisms with oxygen-dependent metabolism and aerobic chemoheterotrophy decreased to 20% in the dysphotic zone, and to 10% in the photic zone. Conversely, during the dry season, the photic zone showed increased representation (15%) compared to the rainy season, considering the oxygen-dependent and aerobic chemoheterotrophy. Regarding manganese oxidation, cave GEM-1423 in the photic zone showed more representatives during the rainy season than the dry season, with a similar trend observed in the dysphotic zone. However, the aphotic zone showed consistently low representation in both seasons. Nitrogen fixation was more prominent in GEM-1423’s photic zone (5%) compared to the dysphotic zone in both seasons, with almost no representation in the aphotic zone. Cellulose degradation in GEM-1423’s photic zone showed a higher number of representatives in both seasons, with a similar number observed in the dry season in the dysphotic zone. The aphotic zone exhibited a low number of representatives in both seasons.

GEM-1462 exhibited a higher number of aerobic chemoheterotrophy microorganisms during the dry season in the dysphotic zone compared to the rainy season, while the aphotic zone showed lower representation in aerobic chemoheterotrophy, with a similar occurrence in the photic zone. In GEM-1423, there was no significant difference in manganese oxidation between the dry and rainy seasons in the photic zone (approximately 5%), whereas the dysphotic zone was more representative in both seasons than the other zones (10%). The aphotic zone showed consistent representation across both seasons (0%). In Cave GEM-1462, the photic zone showed a slight difference in representation of nitrogen fixation microorganisms between the rainy and dry seasons (approximately 5%), while the dysphotic and aphotic zones showed no representation. In GEM-1462’s photic zone, representatives involved in cellulose degradation (cellulolysis) accounted for around 5% in both seasons, while the dysphotic zone exhibited fewer representatives in both seasons. Interestingly, the aphotic zone in the dry season showed more representatives than in the rainy season.

Certain characteristics were predicted in a few samples, such as the ability to degrade urea, observed only in GEM-1423’s photic zone in both seasons. Degradation of aromatic and non-aromatic hydrocarbons occurred only in GEM-1462’s dysphotic zone during the dry season. Specific capabilities, such as fermentation, nitrate and nitrogen respiration, nitrate reduction, chitin degradation, and the presence of pathogenic bacteria, were observed only in the aphotic zone of GEM-1423 during the rainy season. Aerobic nitrate oxidation and nitrification capabilities were observed in the aphotic zone of GEM-1462 during the dry season.

## 4 Discussion

### 4.1 Patterns of microbial diversity in caves

Exploring microbial communities within caves has revealed a complex and varied bacterial landscape, highlighting substantial differences in composition and diversity across different cave zones and seasons. The focus on the surface layer (0–10 cm) represents a methodological decision based on the well-established observation that this soil zone typically harbors the highest microbial activity and is most responsive to environmental and anthropogenic influences (Fierer et al., 2003; Bahram et al., 2018). Standardizing sampling at this depth enabled consistent comparisons across all sites while targeting the most ecologically dynamic portion of the environment. However, vertical stratification of microbial communities is a recognized phenomenon, with deeper layers often hosting distinct taxonomic and functional assemblages due to gradients in oxygen, nutrient availability, and mineral composition (Tripathi et al., 2019; Hu et al., 2021) and possibly in caves.

Identifying a broad spectrum of phyla, including Proteobacteria, Firmicutes, and Euryarchaeota, in both caves underscores the ecological richness and microbial diversity present in these subterranean environments. The data suggest that the caves have few dominant genera, with the three main ones (*Bacillus*, *Acidothermus*, and *Mycobacterium*) (Supplementary Figure 2) being responsible for the decomposition of the organic matter, the metabolization of nitrogenous material and *Acidothermus* cleaving recalcitrant organic matter (cellulose). With this, it is possible to indicate that cave GEM-1423 exhibited higher equitability of bacterial communities than GEM-1462 (higher Shannon index) and, consequently, lower species dominance. Notably, the distinct bacterial profiles between the two caves suggest a strong influence of cave-specific environmental conditions on microbial community diversity. Our results also indicate a relationship between microbial diversity and cave zones, with the photic zone exhibiting a higher richness and equitability of genera compared to the aphotic zone. This difference is strongly related to the selective influence of light, which acts through varying degrees of light availability and consequently influences microbial colonization and community dynamics (Hedrich and Schippers, 2021; Nikolic et al., 2020).

Among the bacteria commonly associated with iron reduction, *Aciditerrimonas* is frequently found in caves or soil environments with high iron density. Although this group was not detected in our samples, other groups capable of iron reduction, such as *Sphingomonas*, *Nocardioides* and *Saccharibacteria*, were observed (Shi et al., 2017).

The beta diversity analysis provides intriguing insights into species turnover dynamics between caves, particularly in response to seasonal variations and differences in light zones. During the dry season, GEM-1462 exhibited significantly higher beta diversity compared to the other cave, indicating greater species turnover between samples collected from different locations within the cave. This suggests that the dry season may exert a stronger influence on microbial community composition in GEM-1462, potentially due to factors such as reduced moisture levels or changes in nutrient availability.

Moreover, differences in beta diversity were observed between the photic and dysphotic zones of the two caves. GEM-1462 demonstrated higher taxon exchange in both the photic and dysphotic zones compared to the other cave, suggesting distinct species turnover dynamics across light zones within caves. These differences may be attributed to variations in environmental factors such as light intensity, temperature, or nutrient availability, which can influence microbial community composition and diversity (Bogdan et al., 2023).

### 4.2 Environmental drivers of microbial diversity and functionality in caves

The distinctive microbial functions observed between caves likely stem from differences in environmental characteristics and resource availability (Reboleira et al., 2022; Zada et al., 2021).

Principal component analysis (PCA) indicated that, although iron (Fe) is geochemically important in the studied environments, its variation was not strongly associated with the main axes of variation in microbial composition. In all panels (Figures 5A–C), the arrow indicates a weak correlation with the PC1 and PC2 axes, suggesting that the variability in iron content among samples is relatively small or that it is not directly associated with the main gradients of microbial community composition. Nevertheless, the proximity of samples from cave GEM-1423 (Figure 5A), the rainy season (Figure 5B) and the aphotic zone (Figure 5C) to the direction of the Fe vector may indicate a punctual or indirect environmental relationship. Compared with the RDA analysis (Figure 7), in which Fe presented a longer and well-defined vector, it is observed that environmental variables may be more ecologically relevant, even when they do not dominate the total variance of the System. Despite sampled points in GEM-1423 indicating a pronounced association with iron concentrations (Figure 5), GEM-1462 may harbor microbial communities specialized in iron-related metabolic pathways, thereby influencing biogeochemical cycling processes and contributing to the adaptation of microbial communities to utilize alternative mineral resources, which in turn contributes to ecosystem resilience (as suggested by Figure 7). Thus, the identified correlation between microbial diversity and iron concentrations in samples from both caves highlights the crucial role of iron in shaping microbial communities within cave ecosystems (Lemes et al., 2021). Fluctuations in iron availability can impact community structure, as different taxa exhibit varying iron requirements or tolerances. Our study showed that GEM-1423 exhibits a lower dominance of genera in the bacterial community compared to GEM-1462, possibly in response to distinct iron concentrations between the caves. Microorganisms capable of utilizing iron for energy metabolism may flourish in environments with higher iron concentrations, thereby influencing community composition (Jiménez et al., 2023; Sireci et al., 2023). For example, the iron-tolerant *Bacillus* genus comprises up to 44% of the bacterial community in GEM-1462 during the dry season. In contrast, in GEM-1423, its higher abundance is observed in the aphotic zone during the rainy season, when the genus reaches a relative abundance of 22%. Furthermore, the taxonomic composition of microbial communities varies between the caves. GEM-1423 harbors a notable presence of Proteobacteria, Planctomycetes, and unique families such as Gemmataceae, whereas GEM-1462 showcases a distinct profile dominated by different phyla and families, such as Bacillaceae, Acidisphaeraceae, Mycobacteriaceae, Pseudomonadaceae and Bradyrhizobiaceae. This variation suggests a less selective environment in GEM-1423 which provides a more diverse array of ecological niches or conditions conducive to supporting a broader range of microbial taxa. In this sense, iron emerges as a critical micronutrient influencing microbial growth and metabolism, thereby limiting the abundance and diversity of microbial communities (Lemes et al., 2021; Liu and Sheng, 2021; Northup et al., 2022). This reinforces our findings that the microbial community in cave GEM-1462 is less diverse and more dominated by certain genera due to exposure to a greater volume of bioavailable iron.

The occurrence of microbial functions varies across seasons and zones, indicating the dynamic nature of ecological processes within cave ecosystems (Ma et al., 2021; Slabe et al., 2021). For instance, in the photic zone during the dry season, energy metabolism functions may predominate, driving the decomposition of organic matter and facilitating nutrient recycling to sustain ecosystem functioning (Ravn et al., 2020). Conversely, in the aphotic zone during the rainy season, functions related to nitrogen metabolism and cellulose degradation may be more pronounced, reflecting nutrient acquisition strategies under nutrient-poor conditions. Energy metabolism in microorganisms encompasses diverse specialized pathways, including aerobic chemoheterotrophy and other chemotrophic processes, which are adapted to environmental gradients. In aphotic and dysphotic zones, microbial communities predominantly rely on the oxidation of organic or inorganic compounds for energy (Ferreira et al., 2020). The limited availability of light in these zones restricts primary production, necessitating dependence on organic matter transported from photic zones or produced locally through microbial processes such as fermentation and anaerobic respiration (Ma et al., 2021). While organic matter may accumulate under certain conditions, microbial activity ensures its decomposition through diverse metabolic strategies (Slabe et al., 2021). The prevalence of chemotrophic pathways highlights the adaptation of microbial communities to environments that lack photosynthetic energy. Iron can be oxidized or reduced by metabolic pathways. The unique roles of microbes, identified by their physiological potential, suggest that communities have adapted to the local environmental conditions within each cave (Paula et al., 2020). The observation of predicted metabolism related to manganese oxidation in cave bacteria indicates their adaptation to the environment, where manganese is abundant in various forms such as manganese oxides on rocks or minerals (Calapa et al., 2021; LaRowe et al., 2021). Moreover, the presence of microorganisms capable of breaking urea in the photic zone of cave GEM-1423 indicates an efficient use of nitrogen, possibly influenced by organic matter or bat populations. Similarly, the capacity to break down hydrocarbons in the dysphotic zone of cave GEM-1462 during the dry season implies adaptation to limited water availability, requiring diverse metabolic capabilities to utilize complex organic compounds. Pathogens and phytopathogens were not observed, although some species of *Mycobacterium* and members of the *Solibacteraceae* are known pathogens (Castelo-Branco et al., 2023; Ramanantsalama et al., 2022), indicating limitations on predictive metabolism.

Seasonal variations have a significant influence on microbial communities inhabiting caves, impacting their diversity and ecological dynamics (Maisnam et al., 2023). Our results indicate that seasons did not alter genus richness but influenced community composition, probably driven by environmental changes in the dry season. Dry seasons, characterized by prolonged droughts, lead to challenges due to decreased moisture levels. This aridity can subject microbial populations to desiccation stress, disrupting their metabolic activity and functionality (Coleine et al., 2021; Surić, 2017). Consequently, certain species may struggle to persist under such conditions, resulting in a decline in overall microbial diversity. The modifications of environmental conditions, especially those pertaining to moisture availability, are of paramount importance in modulating the microbial diversity in subterranean habitats (Bogdan et al., 2023; Ghezzi et al., 2022). Reduced humidity during dry season results in significant impediments, potentially reducing microbial heterogeneity, whereas the rainy periods provide a window for microbial assemblages to flourish and possibly augment their diversity (Kost et al., 2023).

The seasonal oscillations in precipitation, particularly the advent of the rainy season, provide a critical influx of moisture that is instrumental in augmenting microbial activity and propagation within cave ecosystems (Morse et al., 2021). This period of increased hydration serves to replenish essential nutrients, invigorate metabolic activities, and establish optimal conditions for microbial proliferation. As a result, the rainy season is often associated with an enhancement of microbial diversity, as it creates an environment that supports the thriving of a multitude of microbial taxa (Zhang et al., 2023).

### 4.3 Biotechnological potential of microorganisms in caves

Next-generation sequencing (NGS) approaches have become crucial in speleological studies for analyzing the microbial diversity in subterranean ecosystems (Kato et al., 2024). Although metabarcoding techniques do not reflect the current metabolic state or activity of microbial communities, they provide a robust indication of the taxa present in the environment (Garg et al., 2024). This information is valuable for guiding subsequent culture-based approaches, including the development of more selective media for isolating target microorganisms and laboratory assays to investigate specific functional traits (Stewart, 2012). Therefore, while caution must be exercised when interpreting potential functions, metabarcoding offers an essential first step in exploring microbial diversity and prioritizing taxa for more targeted functional and biotechnological studies (Stewart, 2012).

Microorganisms in cave ecosystems have adapted to unique environmental conditions, often involving interactions with minerals, mobilization of inorganic compounds, oxidation of methane and hydrogen, and deriving energy through the hydrolysis of complex and recalcitrant macromolecules (Barton and Jurado, 2007). The high competition for limited resources in these oligotrophic environments drives natural selection, fostering innovation and diversification among bacterial communities (Schluter, 1996). The potential for biotechnology derived from the microbial diversity in caves is vast, encompassing applications that leverage the unique physiology of these microorganisms. For example, hydrocarbon-degrading bacteria could be utilized for environmental remediation, such as cleaning up fuel or oil spills (Das and Chandran, 2011). Microbial pathways for nitrate reduction and nitrogen oxidation offer promising applications for enhancing soil fertility (Dong et al., 2021; Garcia-Segura et al., 2018). Additionally, manganese oxidation by specific bacteria may possibly help mitigate contamination in environments with high levels of toxic metals, while cellulolysis and ureolysis could be harnessed to reduce industrial costs for lytic enzyme production. The genera observed in both caves, including *Acidibacter* and *Acidiphilium*, are known for their metabolic capabilities, particularly in reducing and metabolizing iron from the environment (Malik and Hedrich, 2022; Lu et al., 2010). This characteristic suggests their potential application in biotechnology, particularly in environments affected by the inundation or runoff of dams contaminated with high levels of iron (Chen et al., 2016). Some species of the genus *Crossiella* are known to produce antimicrobial sesquiterpenoids and pyrazine compounds, which exhibit therapeutic activities (Ortiz-López et al., 2023; Yang et al., 2024). Similarly, *Bacillus* are recognized for their diverse activities, including biocontrol against plant pathogens, production of proteases for feather degradation, and roles in cadmium biosorption and phytoremediation (Debez et al., 2024; Mankge et al., 2024; Zainab et al., 2024).

The genus *Rhodanobacter* was predominantly observed in both caves and is known for possessing a diverse set of metal resistance genes, suggesting a correlation with the presence of iron within the cave environment. It is abundant in environments where iron serves as the sole electron acceptor, indicating a metabolic pathway that involves the reduction of iron to produce energy (Huang et al., 2019; Zheng et al., 2024). GEM-1462 interestingly exhibited an increase in *Pseudomonas* populations during the dysphotic zone, particularly during the dry season. This genus is known for its ability to degrade various organic compounds, including aromatic and non-aromatic hydrocarbons, in addition to producing antimicrobial compounds.

The genus *Acidisphaera* is involved in denitrification processes. It exhibits the ability to interact with metals like uranium, in this case, the bioreduction of soluble U(VI) to insoluble U(IV) decreases the mobility of uranium in the environment. In our study, *Acidisphaera* was found in all zones and seasons, except for the photic zone in GEM-1423. Its abundance was highest in the aphotic zone of GEM-1423 during the rainy season. These bacteria are known to be heterotrophic acidophiles, particularly thriving in environments with abundant iron as a metabolic substrate. This suggests a potential link between the aphotic zone and the activation of metabolic pathways in *Acidisphaera* communities, with iron serving as a key substrate. Additionally, the dry season might influence population increases (Bastian et al., 2009; Johnson, 2012; Korzhenkov et al., 2019; Nkongolo et al., 2022). *Acidisphaera* species were identified in environments with high temperatures (30–90 °C, as observed in Yellowstone National Park) and acidity. In Brazil, strains of *Acidisphaera* were isolated from cave environments in Minas Gerais. Furthermore, these bacteria are capable of horizontal gene transfer, potentially facilitating the spread of resistance genes to toxic metal ions found in groundwater (Hemme et al., 2016; Prakash et al., 2012; Sousa et al., 2013).

*Janthinobacterium* species, found in cave environments, exhibit biotechnological potential including hydrocarbon biodegradation, biosurfactant production, efficient nitrogen removal, and antimicrobial activity against multidrug-resistant Gram-negative bacteria (Asencio et al., 2014; Yang et al., 2018; Yap et al., 2024). In summary, the microbial diversity in cave ecosystems offers a valuable reservoir of metabolic capabilities shaped by adaptation to extreme and resource-limited conditions. These unique traits present promising opportunities for applications in environmental remediation, agriculture, industrial biotechnology, and medicine. Further investigations into these microbial communities are underway to better understand and harness their biotechnological potential.

## 5 Conclusion

The differences in microbial diversity among the caves are evident, indicating variations in the observed communities, as well as diverse physiological potentials and interactions with minerals. However, it is noteworthy that all caves exhibit a high presence of iron, suggesting a significant role of this element in shaping microbial ecology within these environments.

The relationship between microbial communities and soil properties, particularly the correlation with iron concentrations in cave GEM-1423, provides insight into the biogeochemical processes that shape these subterranean ecosystems. These results open multiple paths for further research, particularly in understanding the ecological roles of these microbial communities in cave ecosystems, their contributions to biogeochemical cycles, and their responses to environmental changes.

Our findings underscore the complexity of microbial adaptation within cave environments, where multiple environmental factors interact to shape microbial communities’ structure and function (Ma et al., 2021; Shen et al., 2022). The variability in environmental characteristics between the caves highlights the complex interplay between abiotic factors and microbial life, pointing out the potential for unique microbial functions and interactions within each cave environment (Chen et al., 2023; Gatinho et al., 2023).

## Data Availability

The raw sequencing data obtained from this study were submitted to the National Center for Biotechnology Information Sequence Read Archive (NCBI SRA) under the accession numbers SAMN46114166 to SAMN46114144 in the BioProject PRJNA1207052.

## Supplementary material

SUPPLEMENTARY FIGURE 1 Relative abundance of microorganisms [Bacteria and Archaea phylum **(A)** and family **(B)**] inhabiting the cave sediments (GEM-1423 and GEM-1462) from different light zones (photic to aphotic) and sampled in dry and rainy seasons.

SUPPLEMENTARY FIGURE 2 Core microbiome analysis showing the prevalence of the most abundant bacterial genera across samples from caves GEM-1423 and GEM-1462 at different detection thresholds of relative abundance.

SUPPLEMENTARY FIGURE 3 Metabolic diversity index for both caves GEM-1423 and GEM-1462 **(A)** and season **(B)**.

SUPPLEMENTARY FIGURE 4 Pearson correlations among more abundant genera and several mineral concentrations for both caves GEM-1423 and GEM-1462.

SUPPLEMENTARY TABLE 1 Characterization of the GEM-1462 and GEM-1423 caves regarding chemical and environmental variables.
