# Supplementary figures and images for: Exploring microbial dynamics in ferruginous caves: taxonomic and functional diversity across seasons and cave zones
Source: Front Microbiol. 2025 Sep 8;16:1619203. doi: 10.3389/fmicb.2025.1619203 (PMC12450956; doi:10.3389/fmicb.2025.1619203)

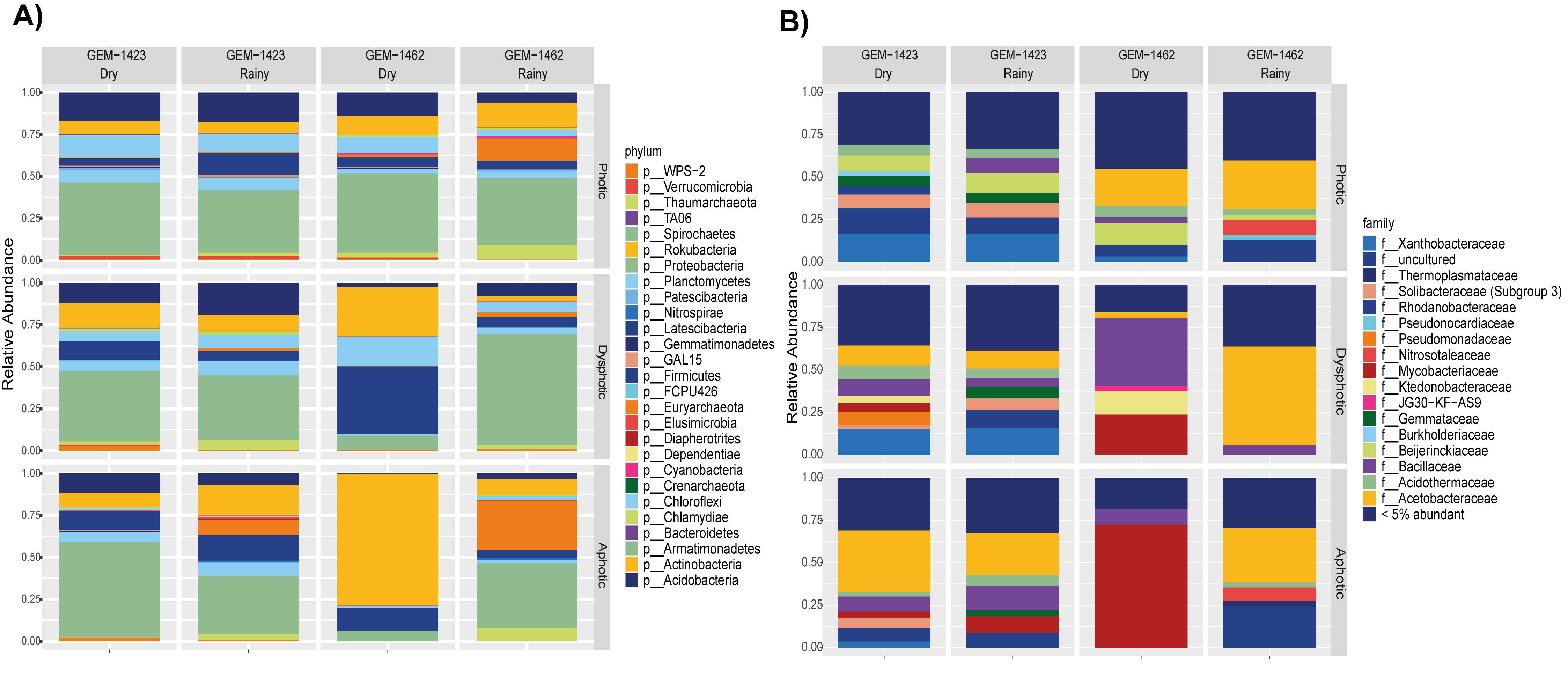

Supplement: SUPPLEMENTARY FIGURE 1 — Relative abundance of microorganisms [Bacteria and Archaea phylum (A) and family (B)] inhabiting the cave sediments (GEM-1423 and GEM-1462) from different light zones (photic to aphotic) and sampled in dry and rainy seasons. [file Image_1.PNG]

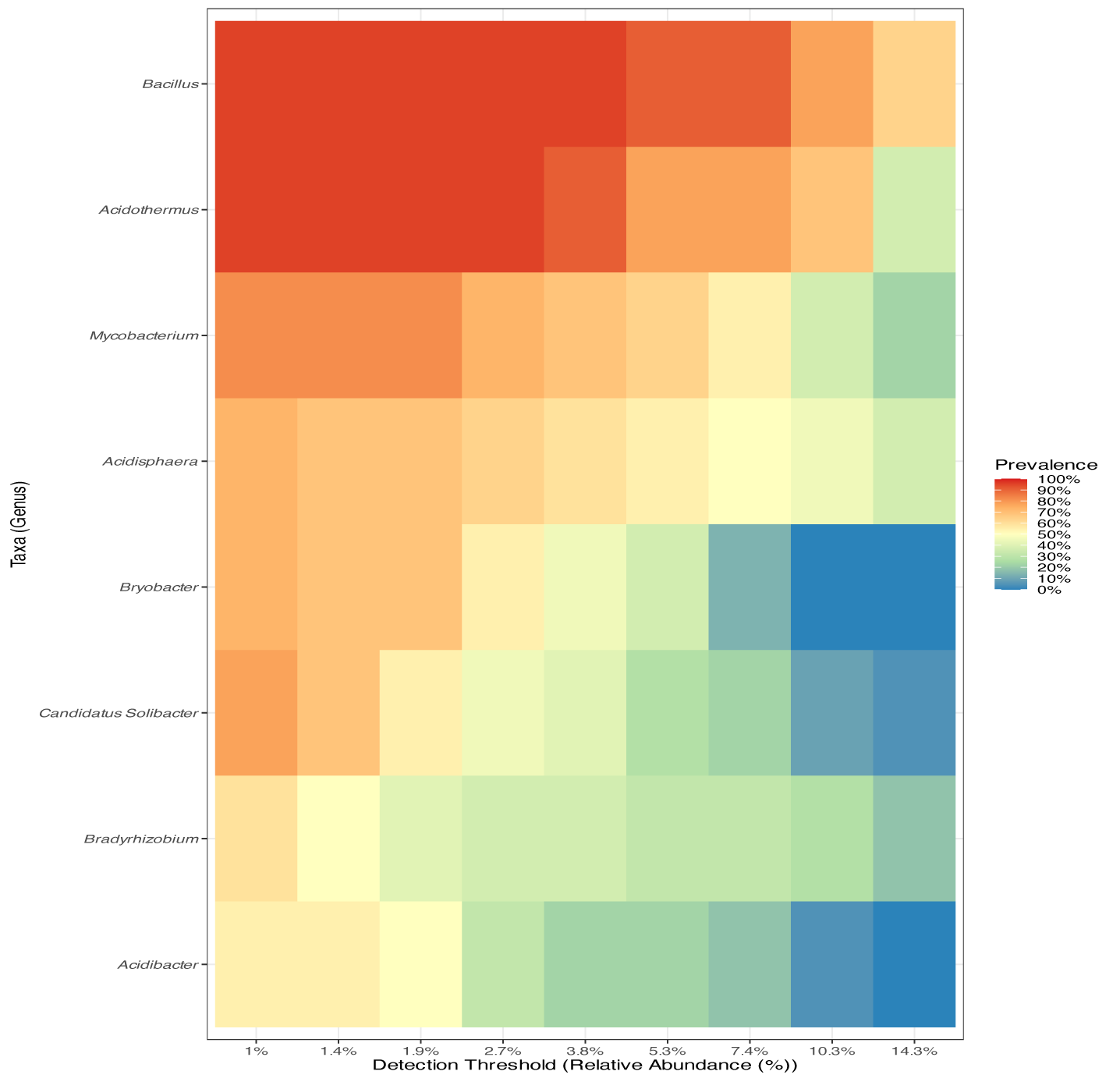

Supplement: SUPPLEMENTARY FIGURE 2 — Core microbiome analysis showing the prevalence of the most abundant bacterial genera across samples from caves GEM-1423 and GEM-1462 at different detection thresholds of relative abundance. [file Image_2.PNG]

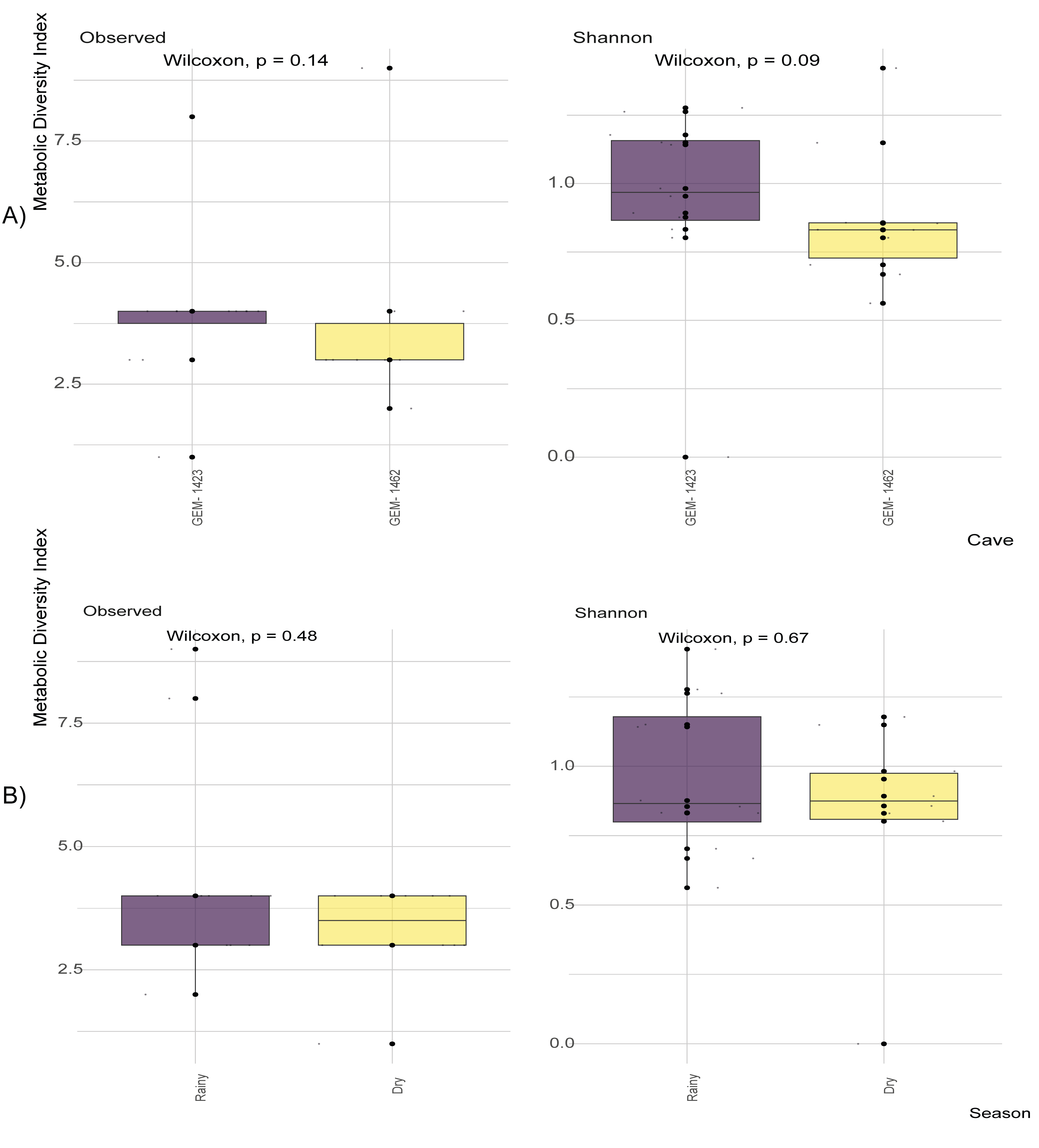

Supplement: SUPPLEMENTARY FIGURE 3 — Metabolic diversity index for both caves GEM-1423 and GEM-1462 (A) and season (B). [file Image_3.PNG]

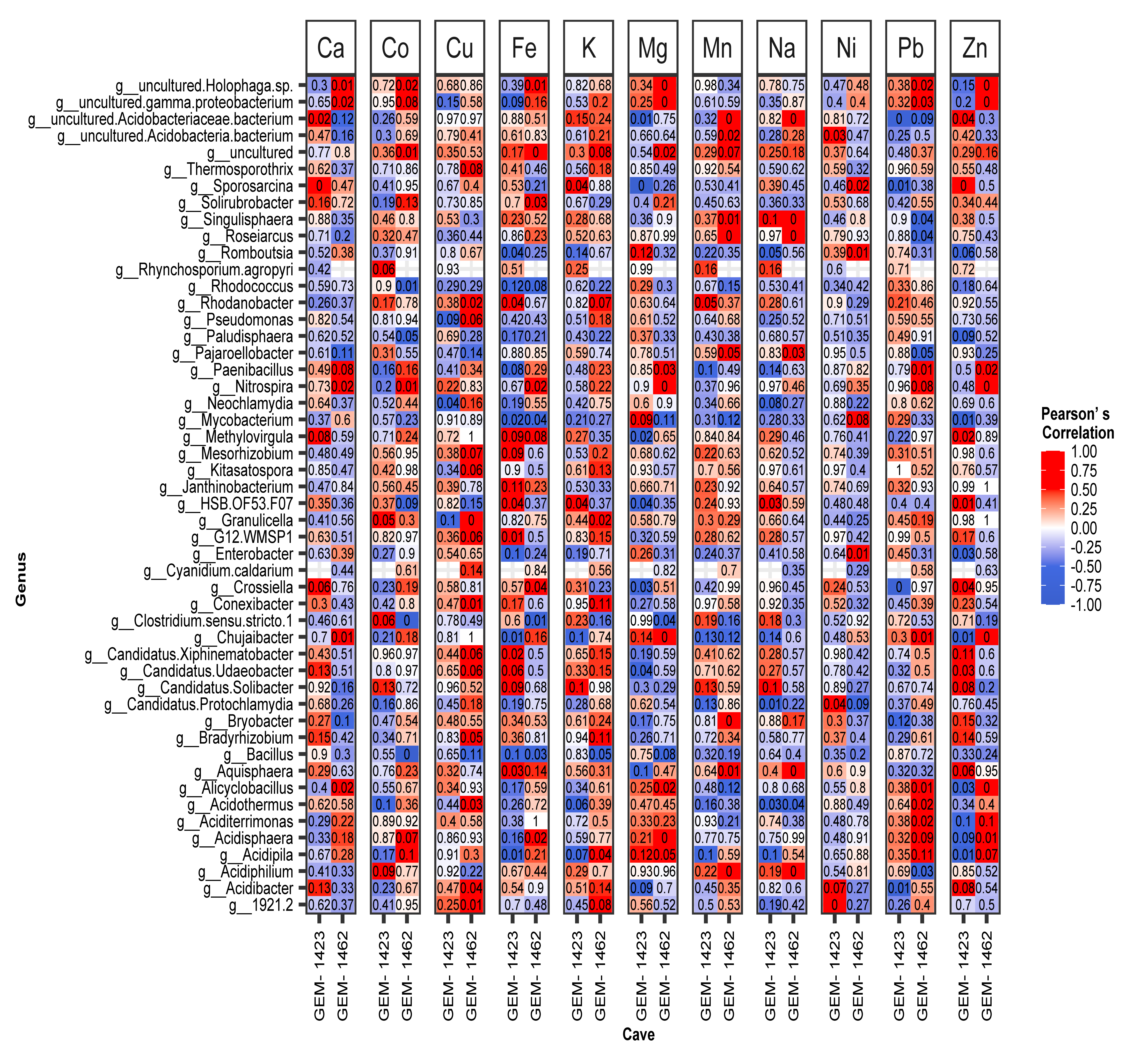

Supplement: SUPPLEMENTARY FIGURE 4 — Pearson correlations among more abundant genera and several mineral concentrations for both caves GEM-1423 and GEM-1462. [file Image_4.PNG]
