# Supplementary material for: Exploring microbial dynamics in ferruginous caves: taxonomic and functional diversity across seasons and cave zones
Source: Front Microbiol. 2025 Sep 8;16:1619203. doi: 10.3389/fmicb.2025.1619203 (PMC12450956; doi:10.3389/fmicb.2025.1619203)
Supplement: SUPPLEMENTARY TABLE 1 — Characterization of the GEM-1462 and GEM-1423 caves regarding chemical and environmental variables. [file Table_1.DOCX]

**Supplementary table 1:** Characterization of the GEM-1462 and GEM-1423 caves regarding chemical and environmental variables.

| **Cave** | **Season** | **Zone** | **Lithology** | **pH** | **Area** | **Volume** | **Slope** | **Height** | **Horizontal Projection** | **Cu** | **Zn** | **Mn** |
| --- | --- | --- | --- | --- | --- | --- | --- | --- | --- | --- | --- | --- |
| GEM- 1462 | Rainy | Aphotic | Jaspilite | 3.98 | 421 | 1035 | 1.8 | 606 | 57.8 | 1085.81 | 155.54 | 700.38 |
| GEM- 1462 | Rainy | Photic | Jaspilite | 3.98 | 421 | 1035 | 1.8 | 606 | 57.8 | 893.83 | 44.78 | 2455.39 |
| GEM- 1462 | Rainy | dysphotic | Jaspilite | 3.98 | 421 | 1035 | 1.8 | 606 | 57.8 | 680.2 | 16.19 | 7177.35 |
| GEM- 1462 | Dry | Aphotic | Jaspilite | 3.98 | 421 | 1035 | 1.8 | 606 | 57.8 | 1233.65 | 13.02 | 311.58 |
| GEM- 1462 | Dry | Photic | Jaspilite | 3.98 | 421 | 1035 | 1.8 | 606 | 57.8 | 1811.31 | 14.56 | 4037.25 |
| GEM- 1462 | Dry | dysphotic | Jaspilite | 3.98 | 421 | 1035 | 1.8 | 606 | 57.8 | 543.1 | 7.85 | 385.27 |
| GEM- 1423 | Rainy | Aphotic | Jaspilite canga | 4.18 | 442.1 | 577 | 2 | 607 | 59.7 | 430.17 | 25.88 | 162.58 |
| GEM- 1423 | Rainy | Photic | Jaspilite canga | 4.18 | 442.1 | 577 | 2 | 607 | 59.7 | 388.96 | 57.87 | 232.31 |
| GEM- 1423 | Rainy | dysphotic | Jaspilite canga | 4.18 | 442.1 | 577 | 2 | 607 | 59.7 | 444.35 | 36.6 | 240.78 |
| GEM- 1423 | Dry | Aphotic | Jaspilite canga | 4.18 | 442.1 | 577 | 2 | 607 | 59.7 | 398.15 | 44.69 | 5630.47 |
| GEM- 1423 | Dry | Photic | Jaspilite canga | 4.18 | 442.1 | 577 | 2 | 607 | 59.7 | 434.39 | 40.58 | 5201.7 |
| GEM- 1423 | Dry | dysphotic | Jaspilite canga | 4.18 | 442.1 | 577 | 2 | 607 | 59.7 | 339.48 | 36.93 | 151.64 |
|  |  |  |  |  |  |  |  |  |  |  |  |  |
| **Cave** | **Season** | **Zone** | **Lithology** | **Ni** | **Pb** | **Cd** | **Co** | **Fe** | **Ca** | **Mg** | **K** | **Na** |
| GEM- 1462 | Rainy | Aphotic | Jaspilite | 57 | 3.6 | 0 | 51.2 | 2E+05 | 5612.5 | 1480 | 350 | 312.5 |
| GEM- 1462 | Rainy | Photic | Jaspilite | 30 | 2.34 | 0 | 48.81 | 2E+05 | 1995.24 | 1120 | 175 | 281.25 |
| GEM- 1462 | Rainy | dysphotic | Jaspilite | 43 | 1.26 | 0 | 42.22 | 2E+05 | 1114.45 | 990 | 312.5 | 406.25 |
| GEM- 1462 | Dry | Aphotic | Jaspilite | 88 | 1.58 | 0 | 38.83 | 1E+05 | 3965.81 | 750 | 237.5 | 281.25 |
| GEM- 1462 | Dry | Photic | Jaspilite | 21 | 2.98 | 0 | 39.7 | 1E+05 | 1154.26 | 870 | 425 | 281.25 |
| GEM- 1462 | Dry | dysphotic | Jaspilite | 18 | 2.32 | 0 | 19.44 | 1E+05 | 1199.05 | 760 | 150 | 281.25 |
| GEM- 1423 | Rainy | Aphotic | Jaspilite canga | 25 | 2.65 | 0 | 30.58 | 2E+05 | 1044.79 | 970 | 100 | 343.75 |
| GEM- 1423 | Rainy | Photic | Jaspilite canga | 21 | 1.36 | 0 | 30.66 | 2E+05 | 1194.07 | 790 | 362.5 | 375 |
| GEM- 1423 | Rainy | dysphotic | Jaspilite canga | 72 | 1.69 | 0 | 30.22 | 2E+05 | 1039.81 | 960 | 112.5 | 312.5 |
| GEM- 1423 | Dry | Aphotic | Jaspilite canga | 20 | 2.56 | 0 | 42.49 | 3E+05 | 1019.9 | 920 | 337.5 | 406.25 |
| GEM- 1423 | Dry | Photic | Jaspilite canga | 34 | 2.94 | 0 | 37.02 | 3E+05 | 1024.88 | 950 | 125 | 375 |
| GEM- 1423 | Dry | dysphotic | Jaspilite canga | 23 | 2.57 | 0 | 32.03 | 2E+05 | 1099.52 | 950 | 125 | 312.5 |
